# Trichosanthin increases Granzyme B penetration into tumor cells by upregulation of CI-MPR on the cell surface

**DOI:** 10.18632/oncotarget.15518

**Published:** 2017-02-19

**Authors:** Chunman Li, Meiqi Zeng, Huju Chi, Jing Shen, Tzi-Bun Ng, Guangyi Jin, Desheng Lu, Xinmin Fan, Bilian Xiong, Zhangang Xiao, Ou Sha

**Affiliations:** ^1^ Department of Anatomy, Histology and Developmental Biology, School of Basic Medical Sciences, Shenzhen University Health Science Centre, Shenzhen, Guangdong, China; ^2^ Laboratory of Molecular Pharmacology, Department of Pharmacology, School of Pharmacy, Southwest Medical University, Luzhou, Sichuan, China; ^3^ School of Biomedical Sciences, Faculty of Medicine, The Chinese University of Hong Kong, Hong Kong, China; ^4^ School of Basic Medical Sciences, Shenzhen University Health Science Centre, Shenzhen, Guangdong, China

**Keywords:** trichosanthin, granzyme B (GrzB), cation-independent mannose-6-phosphate receptor, tumor cells, immunotherapy

## Abstract

Trichosanthin is a plant toxin belonging to the family of ribosome-inactivating proteins. It has various biological and pharmacological activities, including anti-tumor and immunoregulatory effects. In this study, we explored the potential medicinal applications of trichosanthin in cancer immunotherapy. We found that trichosanthin and cation-independent mannose-6-phosphate receptor competitively bind to the Golgi-localized, γ-ear containing and Arf-binding proteins. It in turn promotes the translocation of cation-independent mannose-6-phosphate receptor from the cytosol to the plasma membrane, which is a receptor of Granzyme B. The upregulation of this receptor on the tumor cell surface increased the cell permeability to Granzyme B, and the latter is one of the major factors of cytotoxic T lymphocyte-mediated tumor cell apoptosis. These results suggest a novel potential application of trichosanthin and shed light on its anti-tumor immunotherapy.

## INTRODUCTION

Trichosanthin (TCS) is a well-known traditional Chinese medicine, which is currently used as a midterm abortifacient in clinics. TCS is composed of a single polypeptide chain with a molecular weight of 27 kDa, and it belongs to the family of plant proteins that inactivate the ribosomes of the eukaryotic cell and are therefore named as ribosome-inactivating proteins (RIP) [1]. Besides the RIP activity, TCS has been found to have other biological and pharmaceutical activities such as anti-tumor, anti-HIV, immunoregulatory effects, etc [1–3]. Clinically, TCS has been reported to inhibit the growth of breast cancer, nasopharyngeal carcinoma, lymphoma, and induce apoptotic cell death [4–6]. However, the strong immunogenicity of TCS limited its use in clinical application [7], thus the functional mechanisms induced by TCS caught our attention.

The entry of TCS into mammalian cells is mediated by low density lipoprotein (LDL) or other receptors and through clathrin-coated vesicles [8, 9]. The exact mechanism as to how TCS escapes the lysosomal degradation and is released into the cytosol is still poorly understood. In the cytosol, TCS interacts with ribosomal proteins such as P0 and P1, although whether the interaction is necessary for its RIP activity remains unknown [10]. Recent findings suggest that the anti-HIV and anti-tumor activities of TCS might not be directly related to the inactivation of ribosomes [11]. Thus, it is essential to identify novel TCS binding proteins in mammalian cells, which could reveal much about the intracellular traffic and biological activities of TCS.

In this study, we report that in HepG2 hepatoma cells. TCS binds to GGA (Golgi-localized, γ-ear containing, Arf-binding) proteins, a group of monomeric clathrin adaptors that mediate the transport of several transmembrane proteins with a DXXLL motif in cytoplasmic tail such as cation-dependent and cation-independent mannose 6-phosphate receptors (CD- and CI-MPRs) and BACE1 (β-site APP-cleaving enzyme1) between the Golgi and endosomes [12–15]. We found that the competitive binding of TCS to GGAs decreased the interaction between CI-MPR and GGAs, which in turn resulted in the relocation of CI-MPR from trans-Golgi network (TGN) and late endosomes to plasma membrane. Upregulation of CI-MPR on tumor cell surface increased the cell permeability to GrzB, the major factor involved in cytotoxic T lymphocyte (CTL)-mediated tumor cell apoptosis. These observations indicate that TCS may sensitize tumor cells to CTLs, and consequently enhance the efficacy of cancer immuonotherapy.

## RESULTS

### TCS binds to GGA proteins

**Figure 1:**
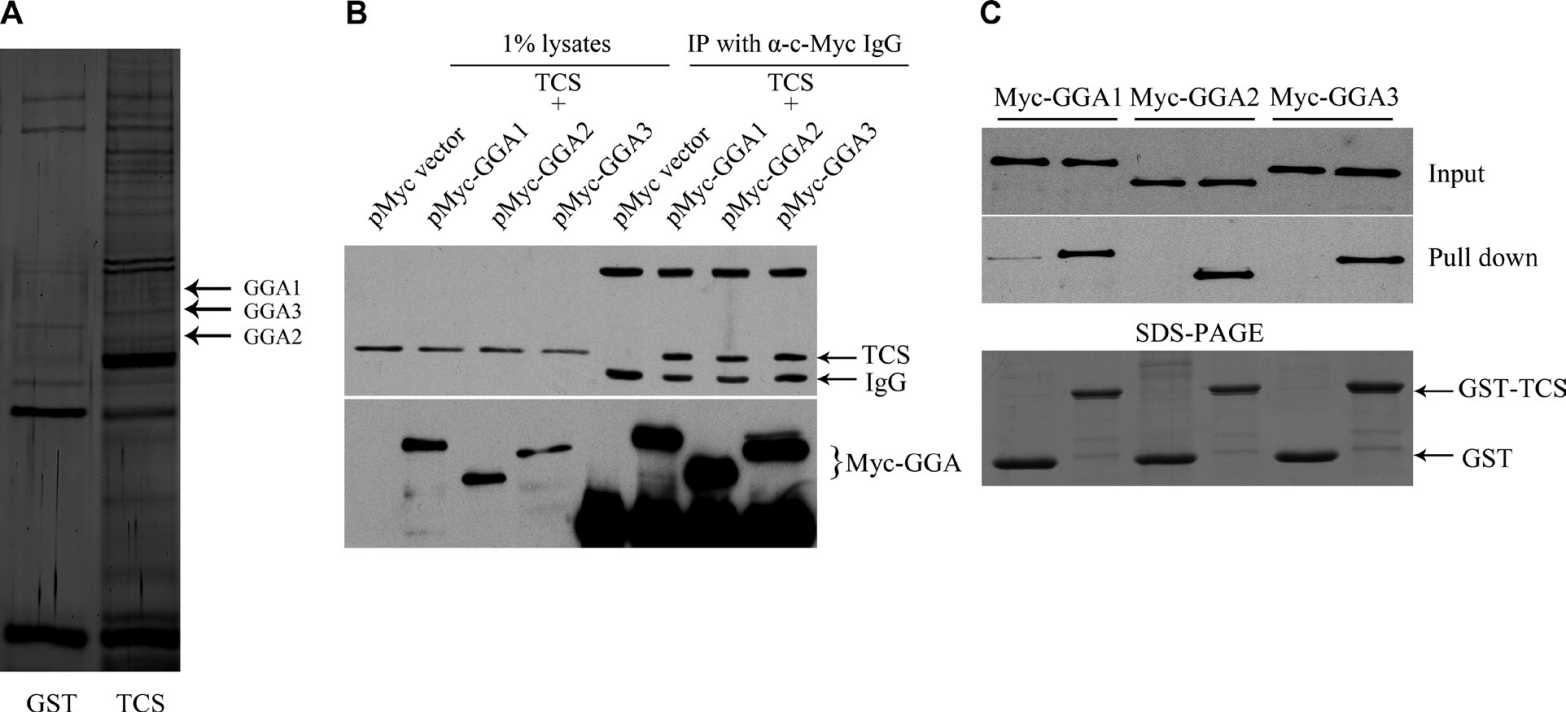
TCS binds to GGA proteins (**A**) Identification and characterization of TCS-binding proteins in HepG2 cells. TCS-associated proteins were pulled down with TCS-Sepharose beads from HepG2 cell lysates using GST-Sepharose beads as a control. The eluted proteins were analyzed by SDS/PAGE and were silver stained. The bands which were detected only in the TCS group were cut and analyzed by LC-MS/MS. GGA proteins showed a higher-percentage of peptide coverage in the corresponding gel slices. (**B**) Myc-GGAs co-immunoprecipitates with TCS protein. HepG2 cells were transfected with the indicated DNA constructs, incubated with TCS and then were immunoprecipitated with anti-Myc antibody. TCS was co-immunoprecipitated with Myc-GGAs. (**C**) GST-pull down assay shows TCS binding to GGA proteins. Glutathione agarose beads were fused with GST or GST-TCS and were incubated with HepG2 cell lysates that were transfected with the indicated plasmids separately. GGAs were bound to GST fusion proteins as detected by the anti-myc antibody.

To identify proteins that interact with TCS, pull down assay was performed on cell lysate from HepG2 cells using CNBr activated Sepharose beads immobilized with TCS or GST as control. The proteins were eluted and separated by SDS-PAGE and then were visualized by silver staining. Candidate bands were excised and analyzed by mass spectrometry. The analysis yielded several members of the GGA family proteins, such as GGA1, GGA2 and GGA3 (Figure 1A). Using an immunoprecipitation (IP) approach, we validated that myc-tagged GGA proteins could co-precipitate with endocytosed TCS (Figure 1B). In the same way, purified GST-TCS fusion proteins were pulled down by Myc-GGAs, whereas GST protein alone was not (Figure 1C).

### TCS interacts with GGA proteins through DIPLL motif in its N-terminal

**Figure 2:**
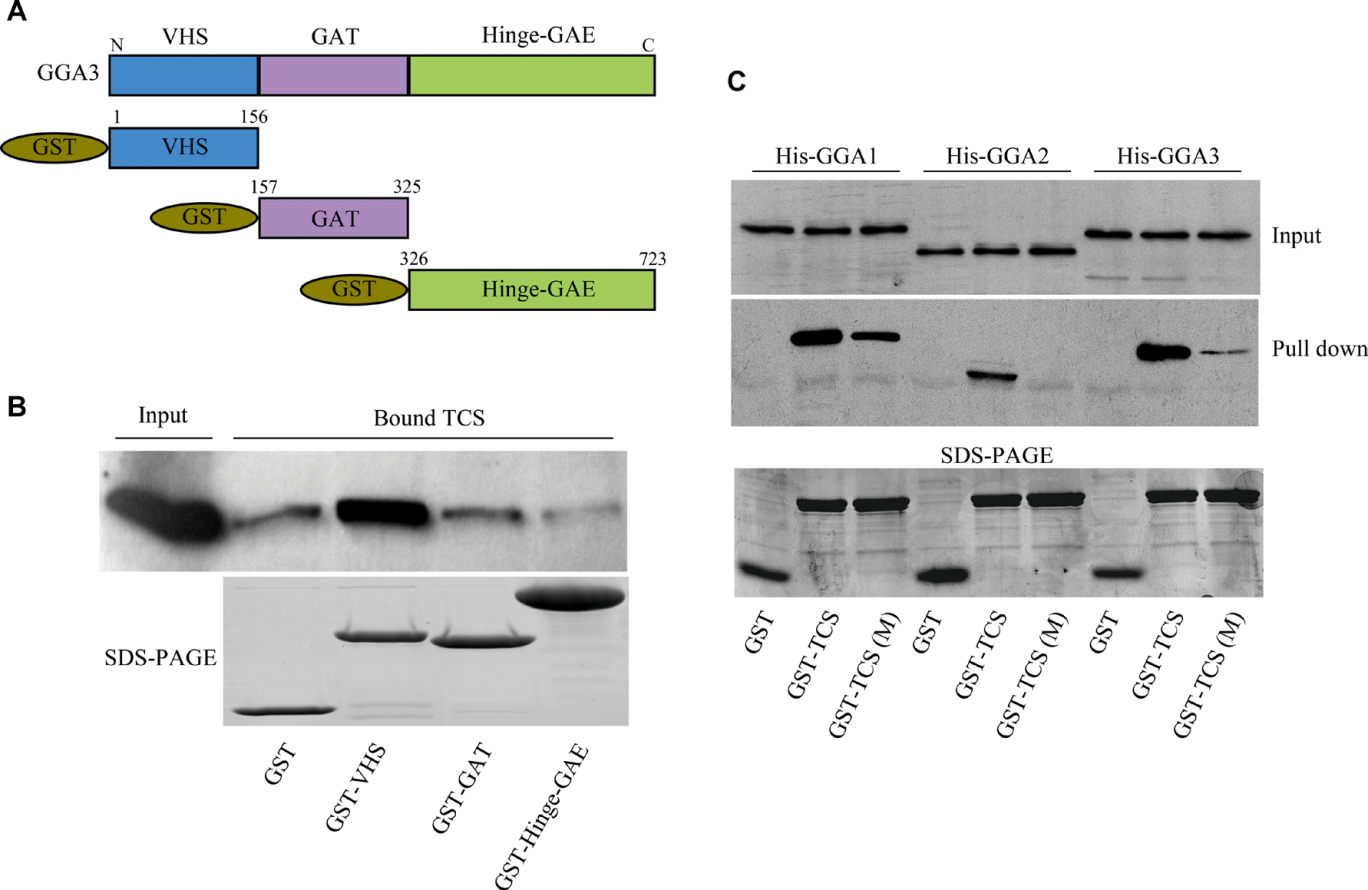
The DXXLL motif in TCS and VHS domain in GGAs mediates their interaction (**A**) Diagram showing the GST-tagged VHS, GAT, hinge and GAE domains of GGA3. (**B**) GST-pull down assays to identify TCS binding domain in GGA3. Bound TCS was detected by immunoblotting using TCS antibody. (**C**) Identification of DXXLL motif in TCS as the GGA-binding site. GST-TCS and its mutant GST-TCS (LL/AA) were conjugated to glutathione agarose beads and then were incubated with purified 6xHis-tagged GGA proteins. The bound GGAs were revealed by immunoblotting using anti-His antibodies and were quantitatively processed.

GGA proteins play a key role in protein sorting between Trans-Golgi and endosomes [16]. All human GGA proteins comprise four structural domains: the N-terminal Vps, Hrs and STAM (VHS) domain that bind to DXXLL motifs of cargo proteins; the GGA and Tom (GAT) domain that are associated with ADP-ribosylation factors (ARF); the γ-adaptin ear (GAE) domain that interact with adaptin and other proteins and the Hinge domain linking GAT and GAE, which binds to clathrin [16–20]. Having confirmed the interactions between TCS and GGAs, we next asked which region of GGA was responsible for TCS binding. For this purpose, the VHS, GAE and Hinge-GAT domain of GGA3 were generated as GST fusion proteins (Figure 2A). By using GST-pull down experiments with different GGA3 domains, we identified that the GGA3 VHS domain is responsible for binding to TCS (Figure 2B). Since several transmembrane proteins interact with VHS domain of GGAs through a DXXLL motif in the cytoplasmic tail, we next precisely analyzed the polypeptide sequence of TCS and found a region in its N-terminus (10-SDDISLLHE-18) in line with this sequence feature [21]. To confirm the acidic dileucine sequence that is responsible for the interaction with GGA VHS domain, we introduced amino acid mutations in leucine residues to alanines (Leu à Ala/Leu à Ala) in TCS and compared the interaction between TCS or TCS (Leu à Ala/Leu à Ala) and GGA3 by the GST-pull down assay. As shown in Figure 2C, the Leu à Ala/Leu à Ala mutations almost completely abrogated the interaction of TCS with GGA2 and GGA3, and strikingly reduced the binding with GGA1. Taken together, these findings indicated that the dileucine motif relative to the N- terminus of TCS has a major effect on GGA binding.

### TCS competitively inhibits CI-MPR binding to GGAs

**Figure 3:**
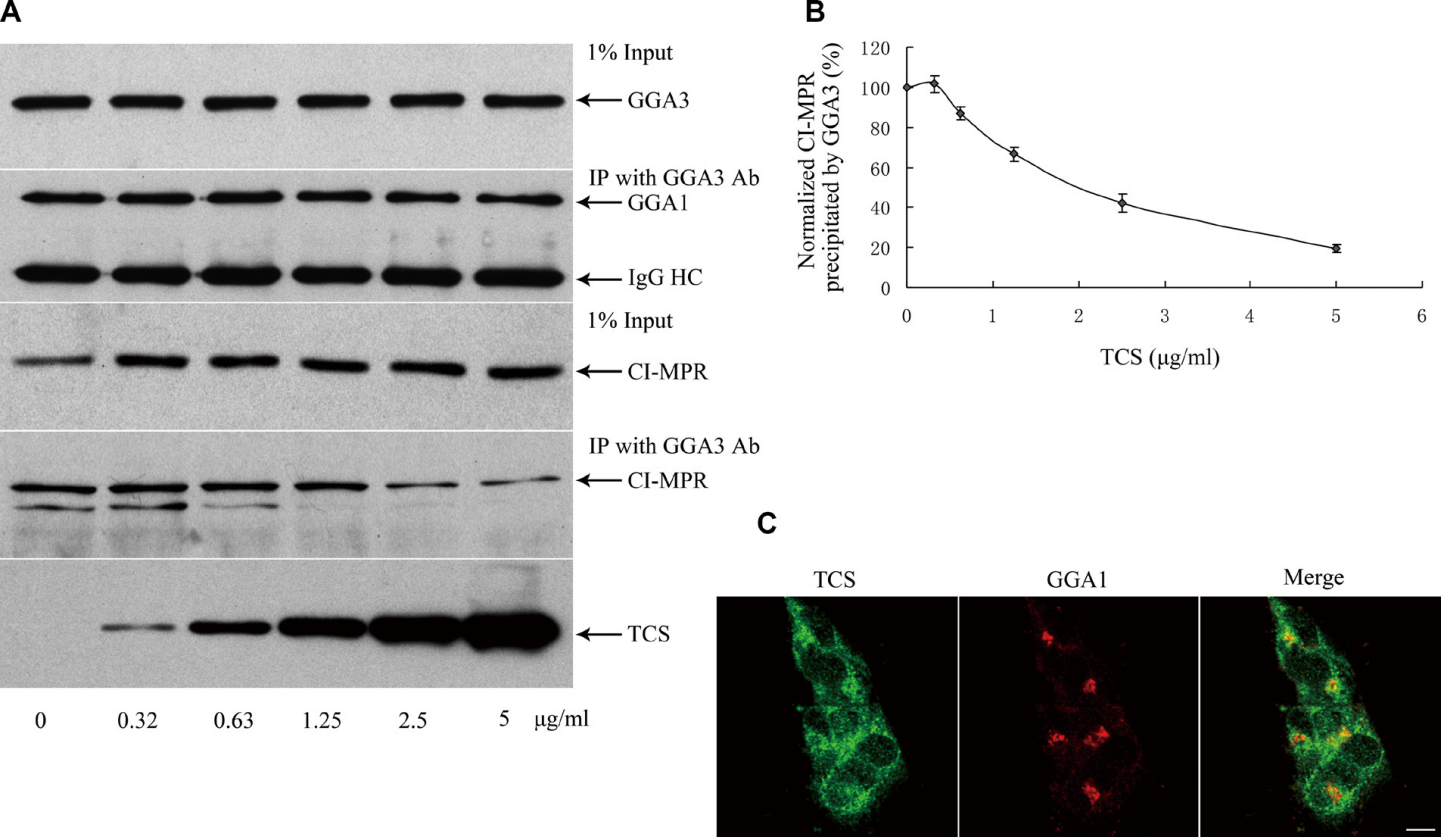
TCS competitively inhibits CI-MPR binding to GGA1 (**A**) Endogenous CI-MPR bound to GGA1 was detected when an increasing amount of TCS was added to HepG2 cells. (**B**) Quantitation of data was shown in panel A (*n* = 3). (**C**) Internalized TCS was partially co-localized with GGA3 in HepG2 cells. Bar =10 μm.

CI-MPR is a transmembrane glycoprotein that delivers newly synthesized lysosomal hydrolases from the TGN to pre-lysosomal compartments [22, 23]. Around 10–20% of the CI-MPR was also found in the plasma membrane [24]. Now that both TCS and CI-MPR bind to the same domain of GGA proteins, we next analyzed whether the interaction of TCS and GGAs would affect the binding of CI-MPR to GGAs. To this end, a competitive co-IP assay was performed using anti-GGA3 antibody in HepG2 cell lysates under increasing load of TCS. As shown in Figure 3A and 3B, GGA3 antibody was able to pull down endogenous GGA3, by accompanying CI-MPR from HepG2 cell lysates, whereas the amount of CI-MPR was recovered by GGA3 beads which were gradually reduced when the amount of TCS was increased. Noteworthily, the expression level of CI-MPR was not significantly affected in the TCS-treated cells. To test whether the interaction between GGA and the proteins that bind to GGA through a domain other than VHS was impaired in the presence of TCS, we also examined the level of clathrin heavy chain pulled down together with GGA3 beads in the above reaction system because clathrin heavy chain binds to the hinge domain of GGA [18]. The results revealed no effect on the interaction between them. Moreover, co-immunostaining for TCS and GGA3 showed that TCS was partially co-localized with GGA3 (Figure 3C). Immunostaining of TGN marker Golgin97 and GGA1 showed that TCS did not change the morphology of the Golgi and the subcellular localization of GGA proteins (data not show). Taken together, these results indicated that TCS can compete with cargo proteins for binding to GGA proteins in the cytosol.

### TCS induces accumulation of CI-MPR on the cell surface

**Figure 4:**
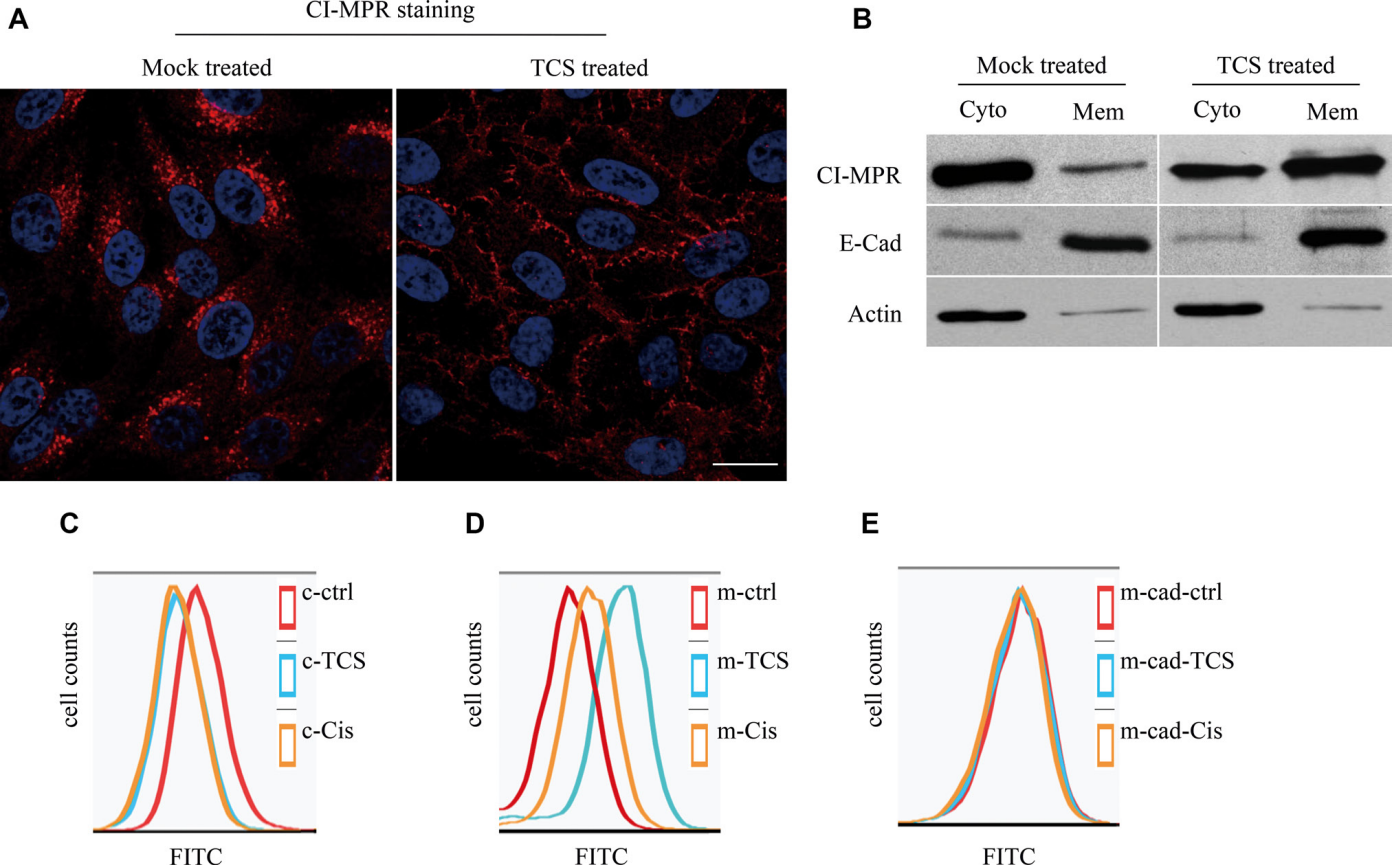
CI-MPR is translocated to cytoplasmic membrane in HepG2 cells after TCS treatment (**A**) Membrane and cytoplasmic fractions were isolated when HepG2 cells were treated with 5 mg/ml TCS or DMSO (as control) and CI-MPR was detected by western blotting. (**B**) HepG2 cells were either treated or not treated with TCS, stained with CI-MPR antibody and then were analyzed by confocal microscopy. Bar = 10 μm. (**C**–**E**) Flow cytometry results show the membrane level of CI-MPR (C) and E-Cadherin (D) or intracellular level of CI-MPR(E) after mock treatment (DMSO only), or treatment with 5mg/ml TCS or 25ng/ml cisplatin (Cis) for 6 hours in HepG2 cells.

Since TCS inhibited CI-MPR binding to GGAs, we next examined whether the transportation of CI-MPR was affected by TCS. In mock treated HepG2 cells, CI-MPR was mostly detected in the perinuclear region, whereas a few punctate structures were also distributed in the periphery. After treatment with 5μg/ml TCS for 6 hours, the perinuclear CI-MPR signal was significantly decreased, and the CI-MPR signal was predominantly located on the plasma membrane and almost could not be detected in the TGN region, although some punctate structures were still in the cytosol (Figure 4A). A similar effect was also observed by Western blot analysis which detected CI-MPR in the cytosol and membrane fraction (Figure 4B). Chemotherapeutic drugs (eg. cisplatin, doxorubicin) have also been found to increase the CI-MPR cell surface expression through autophagy activation [25–27]. Therefore, cisplatin was used as a positive control to compare with the effect of TCS. HepG2 cells were pretreated with TCS or cisplatin for 6 hours and then were fixed and stained with CI-MPR antibody. The presence of CI-MPR on the cell surface was detected by flow cytometry. Compared with BSA-treated mock cells, treatment of cells with TCS increased the cell surface CI-MPR level about 4-fold, and an about 2-fold increase was obtained from cells treated with cisplatin (Figure 4C). While another plasma membrane protein, E-Cadherin, was not affected, which indicated that plasma membrane translocation of CI-MPR induced by TCS and cisplatin was specific (Figure 4D). Accordingly, the amount of CI-MPR in cytosol was decreased in TCS or cisplatin treated cells (Figure 4E).

### TCS increases permeability of tumor cells to GrzB

**Figure 5:**
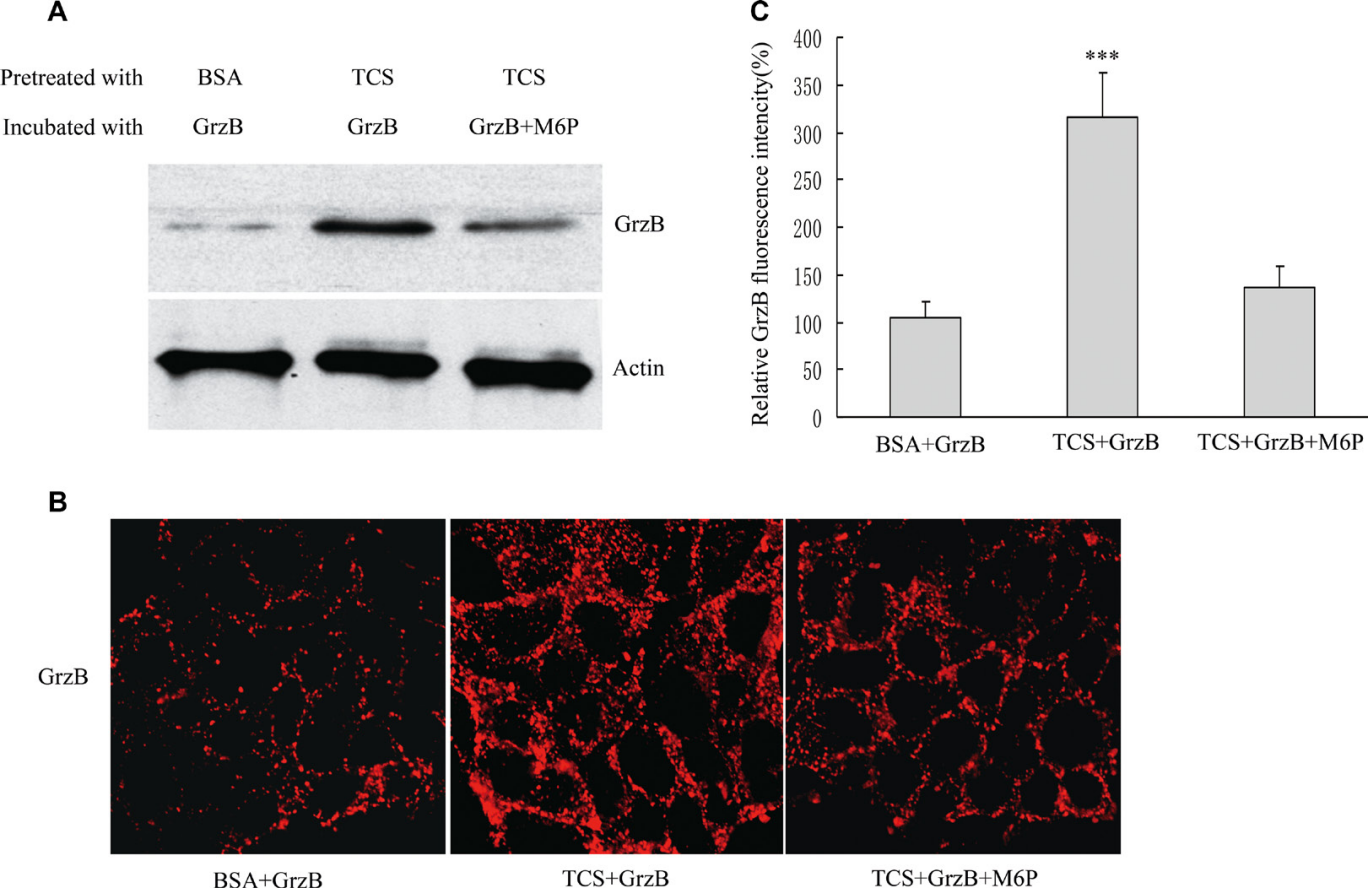
TCS treatment promotes GrzB internalization HepG2 cells were pretreated with BSA (as control) or TCS for 6 hours and then incubated with GrzB or GrzB and M6P. (**A**) Western blotting analysis to detect GrzB in HepG2 cells after various treatments. (**B**) Confocal analysis of GrzB internalized into HepG2 cells after the same treatment as in (A). Bar = 10 μm. (**C**) The average fluorescence intensities (B) were quantified (*n* = 100); *** refers to GrzB values significantly different from control (*p* < 0.001).

The serine proteinase GrzB is one of the critical effectors released by CTLs to induce apoptosis in target cells [28]. Since CI-MPR has been proposed as a death receptor for GrzB binding and uptake, the amount of CI-MPR on tumor cell surface was positively correlated with apoptosis that was mediated by GrzB [29, 30]. We next examined whether TCS prompts GrzB internalization and cell apoptosis. Firstly, we used an inactive form of recombinant mouse GrzB, which did not cause cell apoptosis, to observe the internalization of GrzB [31, 32]. HepG2 cells were pretreated with TCS (5mg/ml) or BSA (5mg/ml) as control for 6 hours and incubated with recombinant mouse GrzB (4 μg/ml) at 37^°^C for 2 hours. As expected, the protein level of GrzB in the cell lysate treated with TCS was significantly higher than the mock treated cell lysate (Figure 5A). We also confirmed the effect of TCS by immunostaining (Figure 5B). Compared with mock BSA-treated cells, TCS treated cells showed an about 3-fold increase in intracellular GrzB level (Figure 5C). In order to further determine whether the increased internalization of GrzB after TCS treatment is caused by the up-regulation of CI-MPR on cell surface, we used soluble M6P to block CI-MPR. TCS-treated HepG2 cells were incubated with GrzB and M6P (20 mM) for 2 hours. As shown in Figure 5A–5C, *in vitro* M6P addition reduced the internalized GrzB in TCS treated cells. These results revealed that TCS prompts GrzB internalization by enhancing the amount of CI-MPR on the cell surfaces.

### TCS enhances *in vitro* apoptosis induced by active GrzB

**Figure 6:**
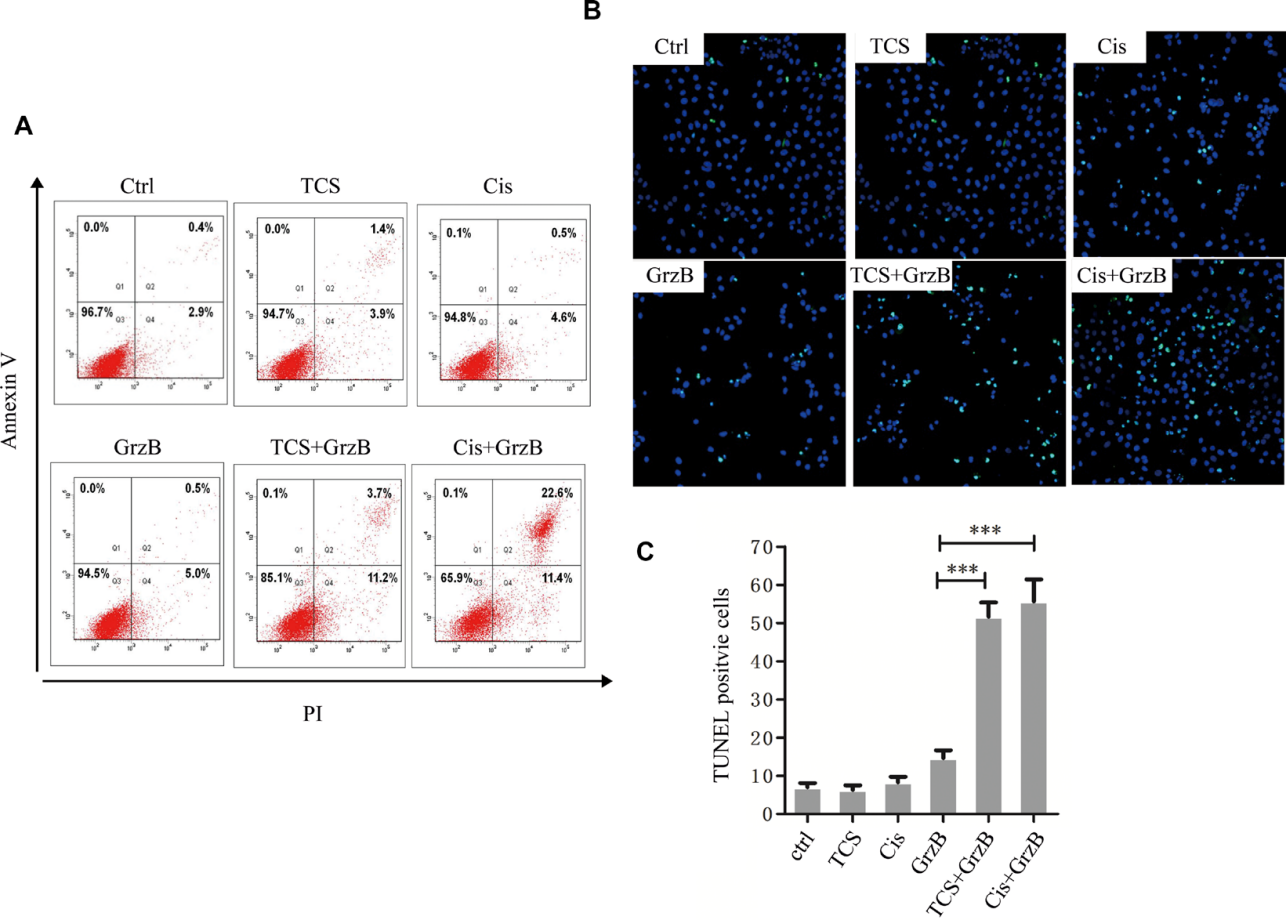
The *in vitro* inhibition of enzymatically active GrzB on cancer cells is greatly increased by TCS (**A**) The cell apoptotic rates were significantly higher in both Cis+GrzB and TCS+GrzB groups. Apoptosis rate was measured by flow cytometry using AnnexinV/PI methods. (**B**) and (**C**), The TUNEL positive signals were greatly increased in cis+GrzB and TCS+GrzB groups (*p* < 0.001) (TUNEL: green; DAPI: blue). Bar = 5 μm.

We next investigated whether TCS promoted apoptosis of tumor cells induced by enzymatically active GrzB. HepG2 cells were first exposed to 5 μg/ml TCS, 25 ng/ml of cisplatin or BSA (control) for 6 hours and then 2 μg/ml of active GrzB or BSA (control) was added, respectively, and incubated for an additional 2 hours. We chose 5 μg/ml as the final concentration of TCS because it markedly increased the amount of CI-MPR on the cell surface but showed no significant apoptosis of the cells. First, HepG2 cells were stained by Annexin-V-FITC and PI and were then subjected to flow cytometry. As shown in Figure 6A, when cells were first treated with BSA for 6 hours and then with GrzB for 2 hours, the apoptotic rates were increased compared to cells treated with only BSA, while a more than 2-fold increase of apoptotic cells was observed in the TCS or cisplatin pretreated group. Furthermore, the percentage of TUNEL-positive cells, induced by GrzB, showed an about 3-fold increase in TCS- or ciaplatin-pretreated group than that of BSA-pretreated group (Figure 6B and 6C).

### TCS promotes GrzB-induced tumor suppression

**Figure 7:**
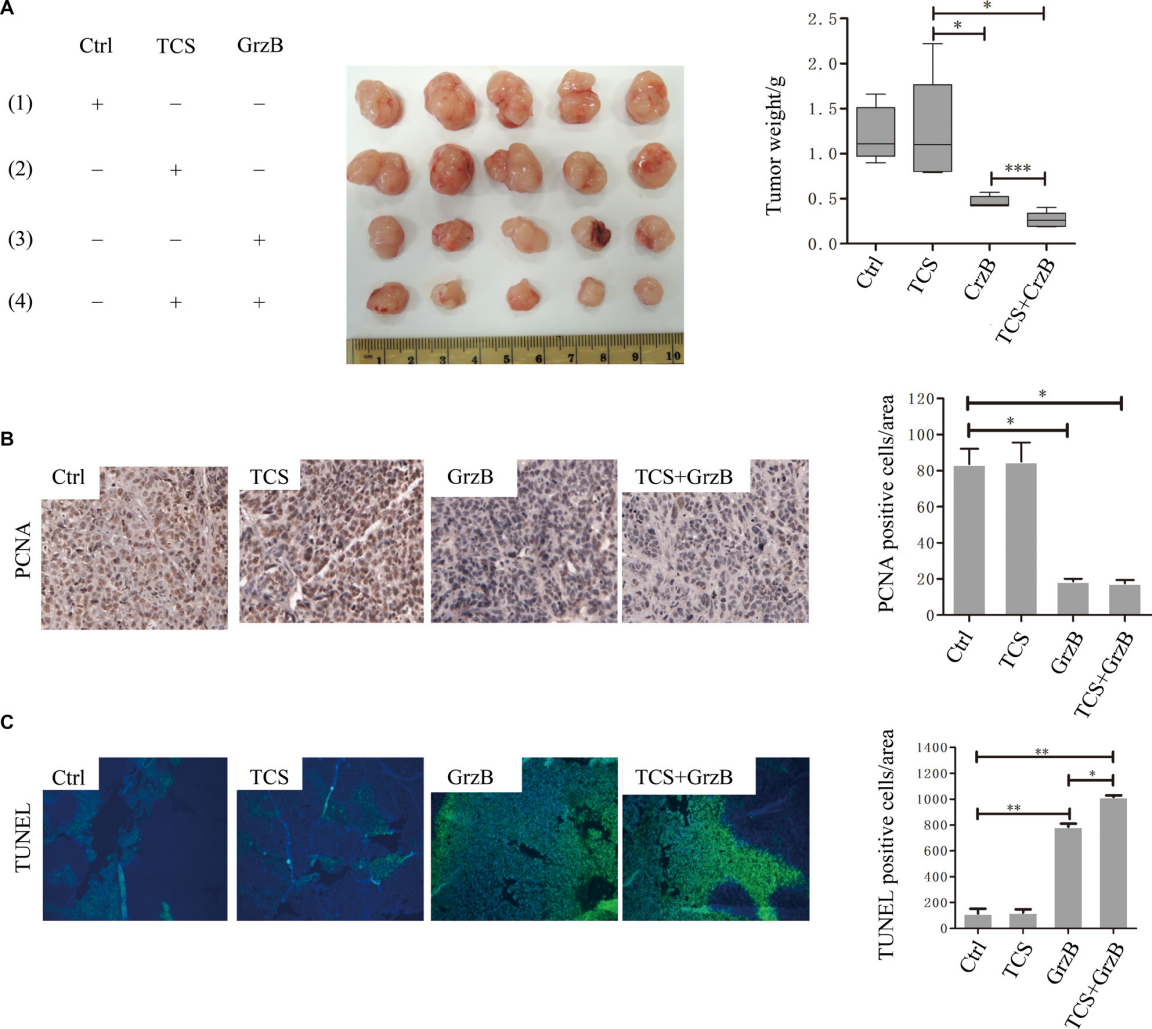
The *in vivo* anti-cancer activity of enzymatically active GrzB is greatly promoted by TCS (**A**) The tumor size was significantly reduced by the treatment of GrzB, and the size was significantly smaller in TCS+GrzB group (*p* < 0.001). (**B**) The positive signals of PCNA were greatly reduced by GrzB, and were much less in TCS+GrzB group. Bar = 50 μm. (**C**) TUNEL positive signals were significantly increased by the treatment of GrzB, and TCS promoted the apoptotic cell death of tumor cells (TUNEL: green; DAPI: blue). Bar = 100 μm.

In order to determine whether TCS also promoted the anti-tumoral activity of human GrzB *in vivo*, we used nude mice injected (i.p.) with HepG2 cells. Compared with the PBS-injected control group, TCS injection almost had no effect. Although GrzB injection only reduced the tumor weight, treatment of mice with both TCS and GrzB more significantly minimized the tumor weight than that of GrzB only (*p* < 0.001; Figure 7A). In addition, immunohistochemical analysis of tumor tissues from each group showed an increased number of TUNEL-positive cells and reduced proliferating cell nuclear antigen (PCNA) signals in TCS+GrzB group compared with the control, TCS only and GrzB only groups (Figure 7B and 7C).

## DISCUSSION

As a ribosome-inactivating protein, TCS exhibited anti-tumor activity in several cancers by inducing apoptosis and by arresting the cell cycle [33]. TCS induced cytotoxicity in a dose-dependent manner. However, the higher doses of TCS caused side effects and antigenicity which limited its use clinically [7, 34]. In the present study, we found that a very low dose of TCS significantly increased the cell surface expression of CI-MPR. CI-MPR has been identified as a death receptor that is critical for GrzB uptake during cytotoxic T cell-induced apoptosis [25, 26, 30], and our findings in turn provided a novel clue for TCS in anti-tumor therapy.

It is well known that GGA proteins mediate transportation of selected cargo proteins (eg.CI-MPR) between TGN and endosomes [18, 19, 35]. Therefore, factors that affect the normal functions of GGA may change the localization of CI-MPR for example, down-regulation of ArfGAP3 by RNAi reduced the membrane association of GGA which in turn resulted in the peripheral dispersion of CI-MPR [36]. Similarly, GGA1 knock down also helped in the translocation of CI-MPR from trans-Golgi to EEA1 positive early endosomes [13]. Therefore, TCS targeting GGA with the same binding site of CI-MPR may mimic the GGA functional loss. In this scenario, more of CI-MPR was transported to the cell surface through the endocytic recycling compartments (ERC).

The cell surface CI-MPR acts as an important factor for GrzB entering into the cells. In addition to promotion of adoptive T cell transfer, recent studies showed that conventional chemotherapeutic agents such as cisplatin and doxorubicin enhanced tumor cell sensitivity by CTL-mediated cancer immunotherapy through upregulation of CI-MPR on the tumor cell surface [25, 26, 37]. Although autophagy may be the possible underlying mechanism in chemotherapy-induced up-regulation of CI-MPR [25, 27], which is different from that of TCS, both drugs resulted in a similar level of increase in the intracellular GrzB when the tumor cells were pretreated. This indicates that TCS, similar to cisplatin, promotes immunotherapy. Therefore, it is worthwhile to further investigate the anti-tumor effect of TCS in combination with immunotherapy.

## MATERIALS AND METHODS

### Reagents, antibodies and plasmids

Reagents and enzymes used in molecular cloning were purchased from Promega. CNBr-Activated Sepharose 4B (cat#17-0430-01) was purchased from GE Healthcare Life Sciences. Pierce^™^ Protein A Agarose beads (cat#20333), Glutathione Agarose beads (cat#G2879), cell culture medium DMEM, fetal bovine serum (FBS) and transfection reagent Lipofectamine^®^ 3000 were from ThermoFisher Scientific. Recombinant Mouse Granzyme B (carrier-free, inactive) was from Biolegend, Recombinant human Granzyme B (active) was from UcallM Biotechology. Unless otherwise indicated, all other chemicals and reagents were purchased from Sigma-Aldrich.

The following primary antibodies were used: monoclonal mouse anti-CI-MPR Ab (CD222, Genetex), mouse anti-cmyc (9E10, Genetex), anti-TCS, anti-GrzB (GB7, Santa Cruz), polyclonal rabbit anti-GGA3 and anti-clathrin (Santa Cruz), anti-TCS (AbCam). HRP-labeled secondary antibodies were from Santa Cruz and Alexa Fluor 488/594-labeled secondary antibodies were from Thermo Scientific.

The ORF of TCS was obtained by RT-PCR using genome extracted from *Trichosanthes kirilowii* Maximowicz by PCR method. The ORF was then digested with the restriction enzymes BamHI and EcoRI and were subcloned in frame into the pGEX-4T-2 vector (GE Health). TCS mutant was generated using QuikChange site-directed mutagenesis (Agilent Technologies). The ORF of GGA1 (NM_013365), GGA2 (NM_015044) and GGA3 (NM_138619) were obtained from human colon mucosa cDNA (Clontech Laboratories) by PCR. The full length GGA1, GGA2, GGA3 and inserted into EcoRI and XhoI sites of pCMVmyc vector (Clontech Laboratories) for expressing myc-tagged recombinant protein in mammalian cells or BamHI and EcoRI sites of pRSET-A vector (Thermo scientific) for Prokaryotic expression. The GGA3 fragments were inserted into pGEX-4T-2 vector for expressing GST fusion proteins.

### Cell culture and transfection

Human hepatocellular carcinoma (HepG2) cells and human cervical cancer (HeLa) cells were cultured in Dulbecco's modified Eagle's medium (DMEM) with 10% fetal bovine serum, 100 units/ml of penicillin, and 100 μg/ml of streptomycin. Transient transfection of these cells was carried out using Lipofectamine^™^ 3000 (Invitrogen) following manufacturer's protocol.

### Cell extraction and TCS-binding assay

To identify TCS binding proteins, 50 μg of TCS or BSA were conjugated with CNBr-activated Sepharose beads according to the manufacturer's instructions. 2 × 10^7^ of HepG2 cells were lysed with 2 ml of lysis buffer consisting of 50 mM Tris-HCl (pH 7.5), 150 mM NaCl, 1 mM EDTA, 1% NP-40 and protease inhibitor cocktails (Roche). The cell lysate was centrifuged at 15,000 g for 10 minutes at 4^°^C. Each 1ml of the supernatant was applied to TCS or BSA conjugated beads and then was incubated at 4^°^C for 2 hours. After washing with lysis buffer five times, the beads were eluted in 1× SDS loading buffer and then boiled for 5 minutes. The beads were briefly centrifuged and then the precipitated proteins were separated by 10% SDS-PAGE and protein bands were visualized by silver staining. The TCS beads precipitated bands were cut from the gel and then subjected to protein identification by mass spectrometry. The proteins were identified by searching in three databases, namely SwissProt, human NCBI and human MSDB, using the Mascot search engine.

### Co-IP and GST-pull down assay

Cells (2 × 10^6^) were lysed with 1ml lysis buffer (50 mM Tris-HCl, pH 7.5, 150 mM NaCl, 1 mM EDTA, 0.1% NP-40 and protease inhibitor cocktails). The cell lysates were centrifuged at 15,000g for 30 minutes at 4^°^C. Then the supernatants were collected for the next step.

For co-IP assays, cell lysates were pre-cleared by protein A agarose beads (Novogen) and then incubated with 1 μg of primary antibody at 4^°^C overnight. Then 50 μl of protein A agarose beads were added and rotated for 1 hour at room temperature. After washing with lysis buffer five times, the beads were eluted with 2× SDS sample buffer and analyzed with SDS-PAGE.

For GST-pull down assays, fragments of interest were subcloned into the pGEX-4T2 vector, and respectively, and were transformed into *E.coli* strain BL21 (DE3). The BL21 clones were grown at 37°C at OD600 of 1.0 and then were induced at 18°C for 16 hours in the presence of 0.1 mM IPTG. Cells were harvested and resuspended in PBS buffer, sonicated and centrifuged at 12,000 g for 30 minutes. The supernatant was incubated with glutathione-Sepharose beads for 1 hour at 4°C. After which, the beads were washed extensively with PBS buffer. Then the beads were incubated with cell lysates for 2 hours at 4°C on a roller. After incubation, the beads were washed with lysis buffer for 5 times. Finally, 40 ml of 2× SDS sample buffer was added and the beads were boiled for 5 minutes for SDS-PAGE and immunoblotting.

### Cell surface protein isolation

The cell surface proteins were isolated using Thermo Scientific^™^ Pierce^™^ Cell Surface Protein Isolation Kit. In brief, about 1 × 10^7^ of HepG2 cells were grown on 150 mm culture dish, washed with ice-cold PBS and biotinylated with 10 ml of the biotin solution for 30 minutes at 4°C. Then 500 μl of quenching buffer was added and cells were washed and then collected. The cell pellets were lysed with 500 μl of lysis buffer and centrifuged. The supernatants were applied to the column containing 500 μl of the NeutrAvidinTM resin. After washing with wash buffer for 3 times, the biotinylated proteins were eluted with 200 μl of 1× SDS sample buffer.

### Flow cytometry

For detection of apoptosis, an Annexin V-FITC/PI Apoptosis kit was used according to the manufacturer's instruction. Briefly, six hours after the drug administration, the cells were washed twice with cold PBS and then were resuspended in the binding buffer (Roche Diagnostics GmbH). 5 μl Annexin V-FITC and 10 μl PI were added, and the cells were incubated for 5 minutes at room temperature in the dark. The cells were finally analyzed by flow cytometry (BD Biosciences FACSCalibur). The measurements were repeated 6 times for each sample.

For CI-MPR or E-cadherin membrane level detection, HepG2 cells were mock treated (DMSO only), treated with 5 mg/ml of TCS or 25 ng/ml Cis respectively for 6 hours. The cells were washed, blocked with 10% FCS and then incubated with CI-MPR (1:100) or E-cadherin (1:50) followed by staining with Alexa568 conjugated Goat anti-Mouse IgG and analyzed by flow cytometry.

For CI-MPR intracellular staining, drug treated HepG2 cells were fixed in 0.01% formaldehyde for 10–15 min, then the membranes were disrupted using 0.5% triton X-100 for 5min followed by staining with Abs and analyzed by flow cytometry.

### Animal model

Male BALB/C nude mice (six weeks old, 15–20 g) from Shenzhen University Animal Services Center were used. All animal experiments were performed with the prior approval of Laboratory Animal Welfare and Ethics Committee, Shenzhen University Health Science Centre. The hepatoma model was developed through subcutaneous implantation of HepG2 cells in mice. Briefly, 1 × 10^6^ HepG2 cells were inoculated subcutaneously on day 0 of each experiment. The tumor bearing mice were divided into four groups with PBS (200 μl; ctrl) injections on days 5, 6, 7, 8, 11, 12, 13 and 14. TCS (2 μg/g body weight) was injected by diluting in 200 μl PBS on days 5, 6, 11 and 12 while PBS was injected only on days 7, 8, 13 and 14. Active GrzB (20 μg/g body weight) was injected by diluting in 200 μl PBS on days 7, 8, 13 and 14 while PBS was injected only on days 5, 6, 11 and 12, or TCS by diluting in 200 μl PBS on days 5, 6, 11 and 12 while active GrzB (20 μg/g body weight) diluted in 200 μl PBS on days 7, 8, 13 and 14 respectively. On day 21, the mice were sacrificed and tumors were collected.

### Immunofluorescence microscopy

The cultured HepG2 cells were fixed in 4% PFA for 15 minutes at room temperature. After fixation, the cells were permeabilized with 0.1% Triton X-100 in PBS for 20 minutes except for staining of CI-MPR on the cell surface, which was permeabilization with 0.05% saponin. The cells were sequentially incubated with blocking buffer (PBS containing 1% BSA, 0.1% gelatin and 0.1% saponin) for 30 minutes, primary antibodies for 2 hours and fluorophore-conjugated secondary antibodies for 1 hour. The coverslips were mounted on glass slides with Prolong AntifadeTM (Invitrogen). Images were captured using an Olympus FV1200 confocal microscope equipped with a 63× objective (NA = 1.4). When two or three markers were imaged in the same cells, each fluorophore was excited and detected sequentially.

### Immunohistochemical staining

Immunohistochemical staining was performed on xenografted tumor tissues with PCNA (1:400 dilution, Cat #: SC-56, Santa Cruz). Subcutaneous tumor tissue sections were incubated with antibodies overnight at 4°C. Mean positive cells were calculated by averaging positive cells from three random fields per slide. The results were also captured by an Olympus FV1200 confocal microscope.

### TUNEL

In brief, six hours after treatment with drugs, the fixed cells or tissue sections were incubated with a mixture of terminal deoxynucleotidyl transferase (TdT) and FITC (Fluorescence *In Situ* Cell Death Detection Kit, Roche Applied Science, Germany). Finally, the cells or tissue sections were observed under a confocal microscope (Olympus FV1200).

### Statistics

All quantitative data were presented as mean ± SEM. The data were analyzed using InStat software (GraphPad Software, USA). The differences were analyzed by using Student's *t* test, and *p* < 0.01 was considered to indicate a statistically significant difference.

## Abbreviations

TCS: trichosanthin
RIP: ribosome-inactivating protein
CI-MPR: cation-independent mannose-6-phosphate receptor
GrzB: Granzyme B
CTL: cytotoxic T lymphocyte
LDL: low density lipoprotein
CD-MPR: cation-dependent mannose-6-phosphate receptor
TGN: trans-Golgi network
IP: immunoprecipitation
VHS: the N-terminal Vps, Hrs and STAM
GAT: the GGA and Tom
Arf: ADP-ribosylation factors
GAE: the γ-adaptin ear
PCNA: proliferating cell nuclear antigen
ERC: endocytic recycling compartments
TdT: terminal deoxynucleotidyl transferase
